# Deciphering maize resistance to late wilt disease caused by *Magnaporthiopsis maydis*: agronomic, anatomical, molecular, and genotypic insights

**DOI:** 10.3389/fpls.2025.1566514

**Published:** 2025-06-03

**Authors:** Walaa R. Abdelghany, Mohsen M. Elsharkawy, Ramy N. F. Abdelkawy, Reda I. Omara, Khaled Abdelaal, Abeer H. Abbas, Wael N. Hozzein, Tarek Essa, Dalal Hussain ALkhalifah, Ayman H. Abou Tabl

**Affiliations:** ^1^ Plant Pathology Research Institute, Agricultural Research Center, Giza, Egypt; ^2^ Agricultural Botany Department, Faculty of Agriculture, Kafrelsheikh University, Kafrelsheikh, Egypt; ^3^ Central Laboratory for Design and Statistical Analysis Research, Agricultural Research Center, Giza, Egypt; ^4^ EPCRS Excellence Center, Plant Pathology and Biotechnology Lab., Faculty of Agriculture, Kafrelsheikh University, Kafrelsheikh, Egypt; ^5^ Botany and Microbiology Department, Faculty of Science, Beni-Suef University, Beni-Suef, Egypt; ^6^ Department of Biology, College of Science, Princess Nourahbint Abdulrahman University, Riyadh, Saudi Arabia; ^7^ Plant Pathology Department, Faculty of Agriculture, Mansoura University, Mansoura, Egypt

**Keywords:** maize genotypes, AMMI, stability, GEI, multi-environments, gene expression, late wilt

## Abstract

**Introduction:**

*Magnaporthiopsis maydis*, the causal agent of late wilt disease (LWD), poses a significant threat to maize production by reducing grain yield and quality. Identifying and developing resistant genotypes adapted to different environments is essential for sustainable crop improvement.

**Methods:**

Fifteen maize genotypes were evaluated for their response to LWD across three growing seasons at two experimental locations—Gemmeiza and Sids. Disease incidence, agronomic performance, anatomical features, and antioxidant enzyme activities were assessed. Gene expression analysis of PR1 and PR4 was conducted using RT-qPCR. Genotype × environment interaction (GEI) was analyzed using combined ANOVA and the additive main effects and multiplicative interaction (AMMI) model.

**Results:**

Significant differences were observed among genotypes, environments, and their interactions (GEI) for disease incidence and yield-related traits (p < 0.05). AMMI analysis confirmed substantial GEI effects on DI% and hundred kernel weight. Genotypes TWC1100, SC30K9, and SC2031 consistently showed the lowest disease incidence and the highest resistance rating index (RRI > 8.3) across both locations, while the susceptible check Boushy recorded the highest DI% and lowest RRI. TWC1100 and SC30K9 also achieved the highest kernel weights at Gemmeiza (42.8 g and 41.5 g, respectively). Stability analysis using AMMI stability value (ASV) identified TWC1100, SC30K9, TWC324, and SC130 as the most stable genotypes. Biochemical analysis revealed that resistant genotypes exhibited higher peroxidase activity and lower electrolyte leakage. Anatomical examination showed superior root structure in resistant genotypes, particularly SC2031. Molecular analysis confirmed the upregulation of PR1 and PR4 genes post-infection, with TWC1100 showing robust expression, while Boushy exhibited minimal gene activation.

**Discussion:**

The integration of agronomic, anatomical, biochemical, and molecular analyses revealed promising maize genotypes with enhanced resistance to late wilt disease (LWD) and stable performance across diverse environments. These findings highlight the potential of these genotypes as valuable candidates for inclusion in breeding programs targeting improved disease resistance and yield stability under varying environmental conditions.

## Introduction

Maize (*Zea mays* L.) is one of the most economically important plants globally, ranking second among cereal crops grown in Egypt in terms of harvested area and production after wheat. There is a gap between the production and consumption of maize in Egypt, which is estimated at approximately 45% (Zohry et al., 2016). Many studies have been conducted to improve the grain yield of maize under various conditions such as drought and weed stress (Majid et al., 2017; El-Sayed et al., 2021a, 2021b). Additionally, many pathogens have harmful effects on maize plants. Late wilt disease (LWD) is the most devastating disease in maize fields, as it lowers crop potential yield and reduces the quality and amount of grain produced (El-Shehawy et al., 2014). Genetic resistance and tolerant maize varieties appear to be the most effective ways to manage LWD and reduce yield loss from disease (El-Shenawy et al., 2022). However, this alternative is restricted owing to the presence of a variety of aggressive strains within *Magnaporthiopsis maydis* populations (Ortiz-Bustos et al., 2016) and, in certain cases, a partial development of resistance that is highly dependent on environmental factors (Abdelghany et al., 2023). The enhancement of quantitative features resistant to LWD may assist in stabilizing maize yield in sensitive areas. However, varied symptom expression in the developed hybrids has been reported, implying variation in disease resistance (Degani et al., 2021). Successful maize production depends on applying production inputs to sustain the environment and agricultural production (Eriksson et al., 2005; Bocianowski et al., 2016). These inputs include adapted cultivars, plant populations, soil tillage, fertilization, insect and disease control, and harvesting (Pandey et al., 2000; Costa et al., 2002; Szulc et al., 2013, 2013). The yield of maize is determined by the genotype and environmental effects, as well as by the genotype × environment interaction (GEI) (Bocianowski et al., 2018; Das et al., 2019).

Under changing climate conditions, crop yield is significantly affected by the growth of genotypes that are unsuitable for the cultivation region. The best method to evaluate the superiority of genotypes is to examine cultivars in various ecological zones and situations to determine their adaptability and stability. The ability of a genotype to provide a consistent yield regardless of environmental influences is known as stability, whereas the ability of a cultivar to produce a consistent and high yield across a variety of environmental circumstances and disease stress is known as adaptability (Kumar et al., 2020). Notwithstanding alterations in the surrounding environment, the performance of a stable genotype remains constant or changes very little (Becker and Leon, 1988). The identification of promising maize hybrids for their adaptability and stability helps in choosing superior maize hybrids for production, which depends on the extent of the GEI. Several stability methods can be used to partition GEI. The additive main effect and multiplicative interaction (AMMI) model is the most commonly used statistical analysis for the interpretation of GEI based on the use of biplots. Evaluation of hybrids across different environments using AMMI analysis helps identify stable hybrids across cultivation ecologies. Genotype × environment studies allow the identification of the ideal location for each genotype, which would maximize grain yield potential and reduce production costs (Oyekunle et al., 2017a; Bocianowski et al., 2024). Bocianowski et al. (2024) examined 69 maize (*Z. mays* L.) hybrids and tested them at five different sites. The AMMI analysis revealed the genotype, environment, and their interactions with grain yield. The analysis of variance revealed that environmental factors accounted for 25.12% of the overall variation in grain yield, genotypic variances accounted for 35.20%, and genotype × environment interactions accounted for 21.18%.

Recent research has demonstrated that pathogenesis-related (PR) proteins accumulate significantly in plants following pathogen infection and are central to plant defense mechanisms (Esmail et al., 2022). Plants use a variety of intricate defensive mechanisms to fend off pathogen attacks, including the synthesis of PR proteins and chemical and structural barriers (Sels et al., 2008). In maize, transcripts of PR genes have been shown to accumulate in response to fungal pathogens such as *Colletotrichum graminicola*, suggesting their involvement in pathogen resistance (Miranda et al., 2017). Among the PR families, PR-1, PR-4, and PR-10 are especially important; they are typically induced during infection and contribute directly to plant immune responses (Li et al., 2011). PR-1 proteins are closely linked to the salicylic acid signaling pathway and are upregulated in resistant maize lines (Ma et al., 2022). PR-4 proteins encoding chitinase from maize exhibit antifungal activity by inhibiting hyphal growth (Bravo et al., 2003), and PR-10 was recently shown to mediate broad-spectrum resistance by modulating defense responses and restricting pathogen colonization (Zhu et al., 2024). The expression of PR genes is also tightly regulated by various plant hormones and chemical inducers, including salicylic acid, jasmonic acid, abscisic acid, benzothiadiazole, and isonicotinic acid, which coordinate the systemic acquired resistance (Sparla et al., 2004; Glazebrook, 2005). Beyond defense, emerging evidence also suggests that PR proteins may play roles in plant growth and developmental pathways (Breen et al., 2017).

The objectives of the present study were to evaluate the anatomical, molecular, and agronomic responses of 15 maize genotypes to *M. maydis* infection, focusing on root structure alterations, expression of resistance genes (*PR1* and *PR4*), and agronomic traits such as disease incidence and yield performance. By analyzing genotype × environment interactions using the AMMI model, the study sought to identify stable genotypes with consistent resistance and high yield across different environments. Ultimately, the goal was to recommend the best-performing genotypes with enhanced resistance to LWD for use in breeding programs, promoting both disease resistance and improved crop productivity.

## Materials and methods

### Plant materials

Fifteen maize genotypes (nine single crosses, five 3-way crosses, and one open-pollinated check cultivar) were grown to study their response to *M. maydis*. Eight hybrids were kindly obtained from the Maize Research Department, Field Crops Research Institute, Agricultural Research Center (ARC), Giza: three from HYTECH Company and three from PIONEER Company. **Table 1** presents the name, origin, and grain color of maize genotypes that were examined.

**Table 1:** Genotype code, name, origin, genetic nature, and grain color of the maize genotypes evaluated in this study.

Genotype no.	Name	Origin	Genetic nature	Grain color
**G1**	SC128	ARC-Egypt	Single cross	White
**G2**	SC130	ARC-Egypt	Single cross	White
**G3**	SC132	ARC-Egypt	Single cross	White
**G4**	SC168	ARC-Egypt	Single cross	Yellow
**G5**	Boushy	Open pollinated	Check	White
**G6**	TWC321	ARC-Egypt	3-way cross	White
**G7**	TWC360	ARC-Egypt	3-way cross	Yellow
**G8**	TWC368	ARC-Egypt	3-way cross	Yellow
**G9**	SC3062	Pioneer-Corteva	Single cross	Yellow
**G10**	SC30K8	Pioneer-Corteva	Single cross	White
**G11**	SC30K9	Pioneer-Corteva	Single cross	White
**G12**	SC2031	HYTECH-Egypt	Single cross	White
**G13**	SC2055	HYTECH-Egypt	Single cross	Yellow
**G14**	TWC1100	HYTECH-Egypt	3-way cross	White
**G15**	TWC324	ARC-Egypt	3-way cross	White

### Inoculum preparation

In a 500-mL glucose glass bottle, 150 g of clean grain sorghum seeds was steeped in water overnight. The next day, the extra water was decanted, and the bottle was autoclaved for 1 hour. Each bottle was inoculated with agar mycelial disc from *M. maydis* culture that had been growing at 27°C for 7 days on Potato Dextrose Agar (PDA) with 0.2% yeast extract (El-Shabrawy and Shehata, 2018). The fungus was allowed to develop for 15 days at this temperature before being detected. Thereafter, the contents of the bottles of each fungal isolate were poured out, and the inoculum from each governorate was divided into two groups: group 1 (Kafr El-Sheikh, Gharbia, and Menoufia) and group 2 (Giza, Beni Suef, and Fayoum) were mixed separately to obtain homogenized inoculum. Afterward, soil infestation was carried out using the mixed inoculum.

### Field experiment

Assessment of resistance levels of 15 maize hybrids to late wilt was conducted in two nursery fields of Agricultural Research Stations, Gemmeiza (Gem.) and Sids (Sid.), Plant Pathology Research Institute, Agricultural Research Centre, Egypt, during the 2022, 2023, and 2024 growing seasons. The Gemmeiza nursery field (30° 79′ 58″ N; 31° 12′ 09″ E) was infested artificially with *M. maydis* group 1, while the Sids nursery field (28° 87′ 63″ N; 30° 88′ 62″ E) was infested by *M. maydis* mixed inoculum group 2. Three replicates of the experiment were run using a randomized complete block design. Each replication consists of four rows that are 6 m long and 80 cm apart, with 20-cm plant spacing. Rows were infested by adding *M. maydis* mixed inoculum of sorghum seeds sown within the rows at two grains per hill and thinned to one plant per hill after 3 weeks from planting. In accordance with a growth regimen suggested by the Maize Research Department at the Field Crops Research Institute, fields were irrigated, treated with pesticides, and fertilized as usual. Sowing of the two tested nursery fields was conducted at the beginning of June, and germination occurred a few days later. The fruit ripening stage in most hybrids was reached 60 days after planting.

### Disease assessment

Disease assessment was based on typical maize late wilt symptoms, including pale green leaves that eventually dry out, resembling water stress, and yellow-brown stems with upward drying symptoms. Disease incidence (DI%) was calculated by dividing the number of infected plants by the total number of plants and multiplying by 100, with monitoring at 76, 83, 90, 97, 104, and 111 days after planting (DAP). DI% was measured when the susceptible check hybrid reached the highest disease level at 115 DAP. The area under the disease progress curve (AUDPC) was determined following Pandey et al. (1989), and the relative AUDPC (rAUDPC) for each genotype was calculated following Akello et al. (2017) as rAUDPC = (genotype AUDPC/susceptible genotype AUDPC) × 100. The relative resistance index (RRI) was derived from the country’s average relative percentage attack (CARPA) on a scale of 0 to 9, where 0 indicates the most susceptible variety and 9 the most resistant variety. The RRI was calculated as RRI = (100 − CARPA)/100 × 9, with a desirable RRI threshold set at ≤7 for resistance (Aslam, 1982; Akhtar et al., 2002).

### Yield parameters

Yield and related traits, i.e., plant height (cm), ear height (cm), ear length (cm), ear diameter (cm), ear weight (g), 100-kernel weight (g) (100 Kwt), and ear yield per plot (kg) (EYP), were estimated.

### Laboratory studies

#### Activities of antioxidant enzymes and electrolyte leakage

For the enzyme assays, 0.5 g of fresh maize leaves was homogenized at a temperature range of 0°C–4°C in 3 mL of 50 mM Tris buffer (pH 7.8), which included 1 mM EDTA–Na_2_ and 7.5% polyvinylpyrrolidone. The resulting homogenates were subjected to centrifugation at 12,000 rpm for 20 minutes at 4°C, after which the enzyme activities were quantified using spectrophotometric methods. The measurements were performed at 25°C with a UV-160A spectrophotometer. The activity of polyphenol oxidase (PPO) was assessed following the methodology outlined by Malik and Singh (1980), while peroxidase (POD) activity was evaluated according to the procedures established by Hammerschmidt et al. (1982). For electrolyte leakage (EL), four individual leaf discs (1 cm^2^) were each placed into flasks containing 25 mL of deionized water. The samples were agitated for 20 hours at room temperature. The initial electrical conductivity was measured using an Acromet AR20 electrical conductivity meter (Fisher Scientific, Chicago, IL, USA). Subsequently, the samples were subjected to a hot water bath at 80°C (176°F) for 1 hour to facilitate cell rupture. Following this treatment, the samples were again placed on the Innova 2100 platform shaker for an additional 20 hours at 21°C (70°F). The final conductivity for each flask was recorded. The percentage of electrolyte leakage was calculated using the following formula: initial conductivity/final conductivity × 100 M (Szalai et al., 1996).

#### Determination of total phenols

Total phenolic content was assessed in the methanolic extract of leaf samples utilizing the Folin–Ciocalteu reagent. In a volumetric flask, 0.1 mL of the extract solution, which contained 1,000 g of extract, was combined with 46 mL of distilled water. Subsequently, 1 mL of the Folin–Ciocalteu reagent was introduced, and the flask was agitated. Following a 3-minute reaction period, 3 mL of a 2% Na_2_CO_3_ aqueous solution was added. The absorbance was then recorded using a spectrophotometer at a wavelength of 750 nm after a 2-hour incubation at 24°C, and the total phenolic content was expressed as micrograms of gallic acid equivalent per gram of dry weight material (Das and Singh, 2016).

#### Biometrical analysis

The experimental design was a randomized complete block design with three replications at all locations and years; a combined analysis of variance across the environments was performed on the basis of individual plot observation using SPSS. Prior to analysis, the data were tested for normality. The test for homogeneity of variances was conducted using Bartlett’s test (Bartlett, 1937). Least significant difference (LSD) values were calculated for the differences between means according to Miller (1981).

#### AMMI model

Stability analysis of the 15 maize genotypes was carried out for grain yield/plant across two environments, representing the combinations of two locations × genotypes × two years according to the AMMI model.

Grain yield data were subjected to multivariate analysis using the AMMI model as described by Gauch and Zobel (1996) to estimate the stability parameters. The AMMI model is as follows.

The AMMI stability value (ASV) is the distance from the coordinate point to the origin in a two-dimensional plot of the first principal component axis (IPCA1) scores (interaction principal component axis 1) against IPCA2 scores (interaction principal component axis 2) in the AMMI model (Purchase, 1997). Because the IPCA1 score contributes more to the G × E interaction sum of squares, a weighted value is needed. This was calculated for each genotype and each environment according to the relative contribution of IPCA1 to IPCA2, as follows:


$$\text{ASVi}=\sqrt{\{\text{SSIPCA}1/\text{SSIPCA}2(\text{IPCA}1\text{ SCORE})\}2+(\text{IPCA}2\text{ SCORE})2\;}$$

where SS_IPCA1_/SS_IPCA2_ was the weight given to the IPCA1 value by dividing the IPCA1 sum of squares by the IPCA2 sum of squares; the larger the ASV value, either negative or positive, the more specifically adapted a genotype was to certain environments. A smaller ASV value indicated a more stable genotype across environments (Purchase, 1997). The AMMI model was performed using the GenStat-v.19. software.

#### Anatomical structure

For anatomical examination, 0.5-cm maize roots were taken at 45 days from sowing and saved in Formalin–Acetic Acid–Alcohol (FAA) for fixation, and then the samples were embedded in paraffin. The cross sections (10–15 μm) were created using a microtome and then fixed on glass slides using Haupt’s adhesive. The slides appeared hollow after xylene treatment, and then double staining with Safranin–Fast green was conducted. The sections were cleared and mounted in Canada balsam. Digital images of roots were recorded using a photomicroscope with a digital camera, and the anatomical characteristics of roots were recorded (Nassar and El-Sahhar, 1998; El-Banna and Abdelaal, 2018; Abdelaal et al., 2019 and Al-Shammari et al., 2024).

#### Quantitative real-time PCR analysis

To identify the expression patterns of PR genes in maize, RNA was isolated from maize leaves using an extraction kit for RNA (Invitrogen, Carlsbad, CA, USA), and then the quantity and quality of RNA were tested by NanoDrop (spectrophotometer, Thermo Scientific, Waltham, MA, USA). Reverse transcription was performed using an RT kit (TakaRa, Maebashi, Japan). Using a Real-Time kit (PrimeScript RT, TakaRa, Japan), 1 μg of RNA was used for first-strand cDNA synthesis. Real-time was carried out using an ABI 7300 Real-Time, Applied Biosystems (Foster City, CA, USA). A final amount of 25 μL was used for each reaction, which contained 400 nM specific primers (**Table 2**), 2.0 μL of diluted cDNA sample, and 12.5 μL of SYBR Green reagent (Master Mix, Applied Biosystems). The GAPDH gene from maize was amplified for internal reference (Kozak, 1999). The 2^−ΔΔCT^ approach was used to determine each gene’s relative mRNA level (Schmittgen and Livak, 2008). At least three similar repetitions of the real-time PCR experiment were conducted. Gene expression data were analyzed for statistically significant differences using Tukey’s tests at a 1% level to compare the means according to Tukey (1949).

**Table 2:** Primers for qRT-PCR of ZmPR-1 and actin genes.

Gene	Forward	Reverse
PR-1	TGGTGTGTTTAGCTCTGGCG	ACGTTCTCATCCCACGACAC
PR4	GCGTTCAAGCCCATCGACA	CGTGTGGGATCACATCCATATAAC
GAPDH	CTTCGGCATTGTTGAGGGTTTG	TCCTTGGCTGAGGGTCCGTC

## Results

### Assessment of genotypic response to disease

Fifteen genotypes were tested for resistance to LWD in Gemmeiza and Sids stations for three growing seasons (2022, 2023, and 2024). Examining the frequency of sensitivity/resistance of genotypes against late wilt, the genotypes showed different reactions to this disease. Data in **Table 3** revealed that the genotypes had significant differences in disease incidence and related traits under two locations (p < 0.05). Generally, the majority of them had a response in resistance to the sensitive range. The highest DI% values at both locations were recorded for genotypes Boushy, SC3062, SC168, SC132, and TWC360. Meanwhile, the lowest DI% was showed by TWC1100, SC2031, SC30K9, SC128, and SC130. However, genotype Boushy showed the highest AUDPC (767.0–860.7) at Gemmeiza and Sids. Boushy recorded the same value (100.00) for rAUDPC and CARPA at both locations and the lowest value for RRI traits at both locations. Meanwhile, genotype TWC1100 recorded the lowest value at both locations for AUDPC, rAUDPC, and CARPA (**Table 3**).

**Table 3:** Late wilt disease parameters of 15 maize genotypes at Gemmeiza and Sids locations, combined over 2022, 2023, and 2024 growing seasons.

No.	Genotypes	DI%		AUDPC		rAUDPC		CARPA		RRI	
		Gem	Sids	Gem	Sids	Gem	Sids	Gem	Sids	Gem	Sids
G1	SC128	8.0	10.8	108.6	128.5	14.2	15.8	13.0	14.5	8.1	8.0
G2	SC130	12.4	6.5	138.7	60.5	18.4	7.0	21.3	8.9	7.4	8.3
G3	SC132	15.3	16.0	175.5	204.7	25.0	25.1	24.9	22.4	7.5	7.4
G4	SC168	22.0	19.5	242.4	223.9	32.3	27.1	36.5	27.8	7.0	7.2
G5	Boushy	60.1	74.4	767	860.7	100.0	100.0	100	100.0	3.7	2.3
G6	TWC321	9.4	13.4	120.7	151.0	16.1	18.1	15.3	18.2	8.3	7.8
G7	TWC360	5.4	21.8	65.5	255.1	8.6	30.9	9.0	28.6	8.4	7.0
G8	TWC368	9.0	12.9	115.7	153.5	15.3	18.8	14.7	18.0	8.2	7.8
G9	SC3062	35.6	38.0	374.9	413.6	48.3	48.6	59.8	50.8	6.1	5.5
G10	SC30K8	11.9	10.8	132.0	124.1	15.8	14.3	18.3	14.8	7.6	8.0
G11	SC30K9	5.5	6.2	67.5	58.0	9.0	6.8	10.6	8.8	8.2	8.4
G12	SC2031	2.9	3.6	28.0	32.6	3.4	4.3	5.0	5.3	8.3	8.7
G13	SC2055	12.7	11.8	159.5	139.6	20.8	17.3	21.5	16.9	7.7	7.9
G14	TWC1100	1.7	3.2	20.7	23.5	3.3	2.7	3.7	4.7	8.8	8.8
G15	TWC324	7.2	12.0	107.2	139.7	13.8	16.1	12.2	15.5	8.0	7.9
LSD		**7.7**	**6.0**	**90.1**	**104.9**	**11.3**	**11.8**	**11.9**	**7.3**	**1.7**	**0.8**

DI%, Disease incidence percentage; AUDPC, Area under disease progress curve; rAUDPC, Relative AUDPC; CARPA, Country average relative percentage attack; RRI, Relative resistance index; Gem, Gemmeiza; LSD, Least significant difference at P<0.05.

For the RRI, genotypes Boushy and SC3062 showed a low value (3.7–2.3) and (6.1–5.5) at Gemmeiza and Sids, respectively, while the genotype TWC1100 exhibited a high value (8.8) at both locations. In all investigations, the epidemiological parameters (DI%, AUDPC, rAUDPC, CARPA, and RRI) of maize genetic resistance to LWD demonstrated that genotypes TWC1100, SC2031, and SC30K9 showed low disease incidence and high RRI of more than 8.3 average for both locations. However, the check cultivar Boushy recorded the highest disease incidence and the lowest RRI (3 average for both locations). Generally, the best genotypes were TWC1100, SC2031, and SC30K9, which showed low scores in disease incidence and related traits (**Table 3**).

### Yield parameters of maize genotypes

Genotypes showed significant differences for all yield studied traits under two locations. Studied yield traits of the Gemmeiza location had the highest value compared to the mean of the Sids location. For plant height, the lowest genotype was SC168, while the highest genotype was TWC1100 average for both locations. Meanwhile, ear height ranged from 93.3–97.9 to 120.8–120.1 cm at Gemmeiza and Sids for SC128 and SC2055, respectively. Even though there is no high difference between genotypes in terms of ear diameter, ear diameter ranged from 4.7 average for both locations to 6.0–5.8 cm at Gemmeiza and Sids. Genotypes TWC1100 and TWC368 showed the highest ear weight at 365.4–367.5 and 317.0–310.7 g at Gemmeiza and Sids, respectively. Likewise, the genotypes had performance variability on ear length. Accordingly, genotype SC132 had the highest mean ear length (23.0–20.9 cm). However, Boushy had the lowest ear length (18.5–18.2 cm) at Gemmeiza and Sids, respectively (**Table 4**).

**Table 4:** Mean yield parameters of 15 maize genotypes at Gemmeiza and Sids locations combined over 2022, 2023, and 2024 seasons.

Genotype	Plant height		Ear height		Ear diameter		Ear weight		Ear length		100 Kwt		Ear yield/plot	
	Gem.	Sids	Gem.	Sids	Gem.	Sids	Gem.	Sids	Gem.	Sids	Gem.	Sids	Gem.	Sids
SC128	234.0	194.0	93.3	97.9	5.0	4.9	320.2	289.4	21.8	20.6	40.0	32.4	5.5	5.0
SC130	219.4	198.8	107.6	103.5	4.9	4.9	298.1	307.2	20.7	20.0	37.5	31.1	6.8	6.2
SC132	207.4	188.4	98.9	96.9	4.7	4.7	283.5	259.1	23.0	20.9	37.9	26.6	4.7	4.1
SC168	203.6	188.3	96.7	99.7	5.0	4.9	281.8	294.9	21.6	20.7	34.0	32.1	5.4	4.9
Boushy	193.3	202.0	95.3	113.1	4.8	4.8	216.2	207.8	18.5	18.2	27.0	24.1	3.5	3.1
TWC321	232.8	214.7	106.9	112.4	4.7	4.7	278.4	275.7	20.4	19.1	38.1	34.6	3.8	3.3
TWC360	223.6	205.7	96.0	98.3	4.9	4.9	266.8	261.5	21.9	20.4	39.1	27.6	4.6	4.0
TWC368	227.3	199.3	109.4	109.2	5.0	5.1	317.0	310.7	21.0	20.4	38.5	28.1	3.6	3.2
SC3062	226.5	222.9	119.0	120.8	4.8	4.7	238.9	230.0	20.0	19.2	33.6	29.2	4.6	4.2
SC30K8	207.2	197.8	101.7	103.8	4.9	4.8	247.5	250.7	18.5	18.7	36.8	29.0	5.9	5.5
SC30K9	228.2	207.1	104.3	104.0	5.0	4.9	301.0	311.0	21.3	20.0	41.5	36.9	6.5	6.0
SC2031	255.8	230.0	111.4	115.5	4.9	5.0	297.6	298.6	21.7	20.2	40.4	37.4	7.8	7.3
SC2055	233.1	216.5	120.8	120.1	4.9	4.9	309.8	315.3	22.1	20.5	37.0	34.6	6.0	5.9
TWC1100	257.4	222.3	114.8	117.0	6.0	5.8	365.4	367.5	20.6	22.0	42.8	36.0	6.1	6.2
TWC324	213.0	212.2	100.3	101.3	4.8	4.6	233.9	236.1	20.2	20.4	38.2	31.6	3.7	3.3
LSD	49.1	29.7	13.0	19.3	0.7	0.6	57.3	37.0	2.8	1.5	2.7	4.3	3.0	1.1

100 Kwt, 100 kernel weight; Gem, Gemmeiza; LSD, Least significant difference at P<0.05.

Significantly higher 100-kernel weight yield means were 40.4–37.4, 41.5–36.9, and 43.8–35.0 g at Gemmeiza and Sids obtained from genotypes SC2031, SC30K9, and TWC1100, respectively. The lowest mean of 100-kernel weight was recorded for Boushy (27.0–24.1 g), followed by genotype SC3062 (33.6–29.2 g) at Gemmeiza and Sids. This difference could be due to the genetic potential of the genotypes. Overall, five genotypes possessed high grain yield: SC2031, SC130, SC30K9, TWC1100, and SC2055 (**Table 3**). The harvested ear yield per plot differed significantly across the genotypes, ranging from 3.5–3.1 kg for Boushy to 7.8–7.3 kg for SC2031 at Gemmeiza and Sids, respectively. Generally, the best genotypes overall studied yield traits were SC2031, SC2055, and TWC1100 (**Table 4**).

### Enzyme activity and electrolyte leakage

Some enzymatic antioxidants of the 15 maize genotypes were determined, i.e., POD, PPO, free phenol, and total phenol (**Table 5**). Peroxidase activity was significantly increased in resistant genotypes SC30K9 and TWC324, as compared to the susceptible check cultivar Boushy.

**Table 5:** Enzyme activities and electrolyte leakage of 15 maize genotypes evaluated to late wilt under field conditions at Gemmeiza and Sids locations.

No.	Genotypes	POD		PPO		T. phenol		F. phenol		Electrolyte leakage	
		Gem	Sids	Gem	Sids	Gem	Sids	Gem	Sids	Gem	Sids
G1	SC128	1.0	1.0	0.2	0.2	1.4	1.6	1.3	1.4	22.4	24.2
G2	SC130	0.4	0.4	0.1	0.1	1.3	1.4	1.2	1.2	20.5	20.7
G3	SC132	1.1	1.1	0.3	0.2	1.2	1.2	1.1	1.0	30.4	30.8
G4	SC168	0.7	0.7	0.1	0.1	1.1	1.2	0.9	1.0	36.3	40.0
G5	Boushy	0.4	0.3	0.1	0.1	1.0	1.3	0.9	1.1	75.4	80.8
G6	TWC321	0.7	0.7	0.1	0.1	1.5	1.3	1.3	1.2	23.1	24.3
G7	TWC360	0.4	0.4	0.1	0.1	1.2	1.3	1.1	1.2	17.8	19.4
G8	TWC368	0.4	0.5	0.1	0.1	1.6	1.5	1.4	1.3	19.5	20.5
G9	SC3062	0.5	0.4	0.2	0.1	1.3	1.6	1.2	1.4	48.7	51.8
G10	SC30K8	2.0	2.0	0.3	0.2	1.2	1.3	0.6	0.7	25.7	26.9
G11	SC30K9	0.8	0.8	0.1	0.1	1.0	1.1	0.4	0.5	23.5	24.6
G12	SC2031	0.6	0.6	0.1	0.1	1.4	1.3	0.6	0.6	13.2	13.3
G13	SC2055	1.5	1.5	0.4	0.4	1.1	1.2	0.5	0.5	21.1	21.7
G14	TWC1100	1.6	1.6	0.2	0.2	1.0	1.0	0.6	0.6	11.2	12.1
G15	TWC324	2.2	2.2	0.2	0.2	1.2	1.1	0.7	0.6	12.3	13.0
	LSD	**0.3**	**0.2**	**0.1**	**0.1**	**0.4**	**0.2**	**0.3**	**0.2**	**4.4**	**4.6**

POD, Peroxidase; PPO, Polyphenol oxidase; F. phenol, Free phenol; T. phenol, Total phenol; Gem, Gemmeiza; LSD, Least Significant Difference at P < 0.05.

However, polyphenol oxidase activity, free phenol, and total phenol were not significantly different in the resistant and susceptible genotypes, as shown in **Table 5**. In contrast, the EL an indicator of membrane permeability was higher in susceptible genotypes than in resistant ones, suggesting that disease resistance is associated with reduced membrane damage.

### AMMI analysis

Combined analysis of variance using the AMMI model established the treatments (T), genotype (G), environment (E), genotype × environment interaction (G × E), and principal component axis (IPCA) items. The results in **Table 6** proved that all AMMI items had significant variances. The findings showed that the (G × E) interaction effect accounted for 4.5% of the variation. However, genotypes and environments explained 90.4% and 0.7%, respectively. The G × E interaction effects in this investigation were divided into IPCA1 (71.6%) and IPCA2 (24.1%), gathering 95.7% of the total G × E interaction (GEI) that could be explained by the two first IPCAs. The AMMI analysis result provides information on which genotype to use in which environment to achieve a decrease in disease incidence.

**Table 6:** Additive Main effect and Multiplicative Interaction (AMMI) analysis of variance for disease incidence (DI %).

Source	d.f.	s.s.	m.s.	Explained %	GEI %
Total	269	74570	277.2		
Treatments	89	71310	801.2	95.6**	
Genotypes	14	67407	4814.8	90.4**	
Environments	5	543	108.6	0.7*	
Blocks	12	486	40.5	0.7**	
Interactions	70	3361	48	4.5**	
IPCA1	18	2406	133.7		71.6**
IPCA2	16	811	50.7		24.1**
Residuals	36	143	4		4.3
Error	168	2773	16.5	3.7	

*Significant at 0.05 probability level; **Significant at 0.01 probability level.

**Table 7** displays the ASV, which ranks the 15 genotypes, as well as the AMMI model IPCA1 and IPCA2 scores for each genotype. As a result, genotypes TWC1100, SC2031, SC30K9, SC128, and SC130 had low disease incidence, whereas TWC321, TWC368, SC128, TWC324, and SC132 were the most stable. Conversely, Boushy, TWC360, SC130, SC168, and SC3062 exhibited instability. This measure is crucial to rank and quantify genotypes based on the stability of disease incidence. According to the genotype selection index (GSI), the least GSI is considered to be the most stable with low disease incidence. Based on the GSI, the most desirable genotypes for the selection of both stability and low disease incidence were TWC1100, SC128, TWC368, TWC321, and TWC324 according to the results of the AMMI biplot and estimated stability parameters (**Table 7**).

**Table 7:** IPCAg1, IPCAg2 scores, AMMI stability value and Genotype selection Index of 15 maize genotypes for disease incidence (DI %).

No.	Genotypes	Mean DI	RDI	IPCAg1	IPCAg2	ASV	RASV	GSI
G1	SC128	9.4	4	0.22083	0.71405	0.969	3	7
G2	SC130	9.5	5	1.82496	−0.99454	5.504	13	18
G3	SC132	15.7	12	0.57172	0.32001	1.726	5	17
G4	SC168	20.8	13	1.26058	−0.09072	3.74	12	25
G5	Boushy	67.2	15	−3.27646	−1.19328	9.792	15	30
G6	TWC321	11.4	9	0.01431	0.77053	0.772	1	10
G7	TWC360	13.6	11	−2.89078	1.76616	8.755	14	25
G8	TWC368	11.0	7	−0.31597	−0.20637	0.96	2	9
G9	SC3062	36.8	14	−0.73447	−2.57762	3.375	11	25
G10	SC30K8	11.3	8	0.79652	−0.64221	2.448	8	16
G11	SC30K9	5.9	3	0.67748	0.52744	2.078	7	10
G12	SC2031	3.3	2	0.90418	1.12135	2.907	10	12
G13	SC2055	12.3	10	0.88237	−0.53471	2.671	9	19
G14	TWC1100	2.5	1	0.58188	0.86869	1.932	6	7
G15	TWC324	9.6	6	−0.51716	0.15123	1.541	4	10

DI, Disease incidence; RDI, Rank of disease incidence; IPCA1, IPCA2, Interaction principal component axes 1 and 2; AMMI, Additive main effect and multiplicative interaction; ASVi, AMMI stability value; RASVi, Rank of AMMI stability value; GSIi, Genotype selection index.

The results of AMMI combined analysis of variance across six environments for the 15 genotypes of 100-kernel weight revealed that tested treatments (T), genotypes (G), environments (E), interaction (G × E), and the IPCA1 were highly significant (p < 0.01) (**Table 8**). Observed highly significant differences between the genotypes represented the difference in the genetic potentiality of the genotypes for the analyzed 100-kernel weight trait; additionally, the observed highly significant differences between the studied environments represent the significant genotype effect in the additive structure of data for the 100-kernel weight among the environments.

**Table 8:** Additive Main effect and Multiplicative Interaction (AMMI) analysis of variance for 100 kernel weight.

Source	d.f.	s.s.	m.s.	Explained %	GEI%
Total	269	7351	27.33		
Treatments	89	6475	72.75	88.1**	
Genotypes	14	3155	225.33	42.9**	
Environments	5	2578	515.64	35.1**	
Block	12	131	10.95	1.8**	
Interactions	70	742	10.6	10.1**	
IPCA1	18	673	37.41		90.7**
IPCA2	16	46	2.86		6.2
Residuals	36	23	0.64		3.1
Error	168	745	4.43	10.1	

*Significant at 0.05 probability level; **Significant at 0.01 probability level.

However, blocks, principal component analysis (IPCA2), and residuals were insignificant for 100-kernel weights (**Table 8**). Approximately 35.1% of the variation in 100-kernel weight was attributed to the tested environments, 42.9% to genotypes, and 10.1% to interaction (G × E) sources of variation. Environments contributed almost three times the total variance compared to the interaction (G × E) and were almost equivalent to genotypes. There are two main components to the genotype × environment interaction (IPCA1 and IPCA2). The IPCA1 accounted for approximately 90.7%, while the IPCA2 accounted for 6.2%; thus, IPCA1 and IPCA2 together accounted for 96.9% of the total G × E interaction (GEI) (**Table 8**).

For 100-kernel weight, the ASVs with their ranking for 15 genotypes in addition to the AMMI model 2 IPCA1 and IPCA2 scores for each genotype are shown in **Table 9**.

**Table 9:** IPCAg1, IPCAg2 scores, AMMI stability value and Genotype selection Index of 15 maize genotypes for 100 kernel weight.

No.	Genotypes	100 Kwt	RYi	IPCAg1	IPCAg2	ASV	RASV	GSI
G1	SC128	36.2	5	0.42764	−0.10643	6.3	5	10
G2	SC130	34.3	8	0.16447	0.48374	2.5	1	9
G3	SC132	32.3	13	1.71559	0.07846	25.2	15	28
G4	SC168	33.0	11	−1.34675	−0.08244	19.8	13	24
G5	Boushy	25.6	15	−0.95786	0.58359	14.1	9	24
G6	TWC321	36.4	4	−0.81234	0.29726	11.9	8	12
G7	TWC360	33.3	9	1.69918	−0.61125	25.0	14	23
G8	TWC368	33.3	10	1.33377	−0.54564	19.6	12	22
G9	SC3062	31.4	14	−0.57947	−0.26479	8.5	6	20
G10	SC30K8	32.9	12	0.60417	0.28259	8.9	7	19
G11	SC30K9	39.2	2	−0.41614	0.52951	6.1	4	6
G12	SC2031	38.9	3	−1.09382	−0.86516	16.1	10	13
G13	SC2055	35.8	6	−1.24782	−0.73747	18.4	11	17
G14	TWC1100	39.4	1	0.33947	0.90825	5.1	3	4
G15	TWC324	34.9	7	0.16991	0.0498	2.5	2	9

100 Kwt, Mean of 100 kernel weight; RYi, Rank of 100 kernel weight; IPCA1, IPCA2, Interaction principal component axes 1 and 2; ASVi, AMMI stability value; RASVi, Rank of AMMI stability value; GSIi, Genotype selection index.

Accordingly, TWC1100, SC30K9, SC2031, TWC321, and SC128 exhibited a high 100-kernel weight, while genotypes SC130, TWC324, and TWC1100 were the most stable. However, genotypes SC132, TWC360, and SC168 were unstable, with corresponding rank ASV values of 15, 14, and 13, respectively. According to the GSI, the most favorable genotypes for the selection of both stability and high 100-kernel weight were TWC1100, followed by SC30K9, SC130, and TWC324, which had the highest estimated stability parameters and AMMI biplot result (**Table 9**).

### AMMI biplot for disease incidence of 15 maize in six environments

**Figure 1:**
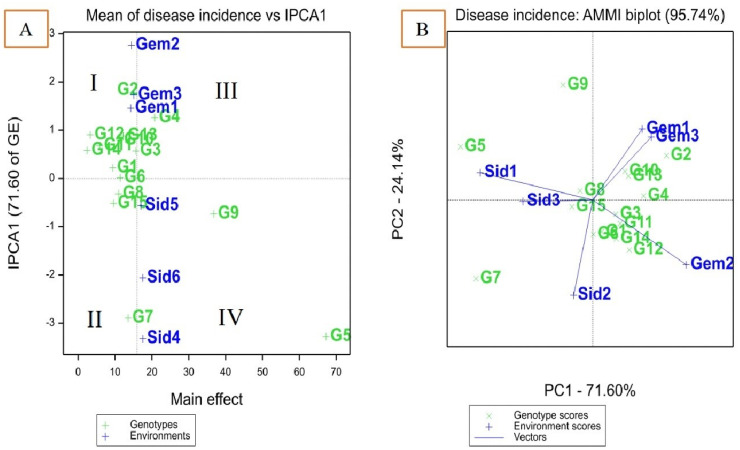
AMMI 1 biplot of interaction principal component axis (IPCA1) against mean effect for disease incidence of 15 maize genotypes in six environments **(A)**, IPCA1 vs. (IPCA2) for disease incidence **(B)**.

AMMI model 1 showed that differences in the main effects are represented by displacement along the x-axis, while differences in the interaction effects are represented by displacement along the y-axis. The environments were divided into four partitions (I, II, III, and IV) as shown in **Figure 1A**. Disease incidence scores were lower for genotypes or environments located on the left side of the y-axis midpoint than for those located on the right. Genotypes TWC321, SC128, TWC368, and TWC324 adapted well to most environments. The disease incidence was lower for these genotypes compared to those on the right side (e.g., SC3062 and Boushy) of the y-axis. High IPCA scores were comparatively displayed by Gemmeiza 1, 2, and 3, which significantly increased GEI. Based on mean disease incidence, these environments were favorable to genotypes with reduced disease incidence. Environments Sids 1 and Sids 2 were the least favorable environment for almost all genotypes with low disease incidence scores and smaller IPCA1 scores (**Figure 1A**). All genotypes gave low infection under the Gemmeiza location except Boushy, TWC360, and SC3062. Genotypes SC2055, SC130, TWC1100, SC30K9, and SC128 had the lowest disease incidence with higher IPCA1 scores than other genotypes. Genotypes Boushy, TWC360, and SC3062 were placed far from the Gemmeiza location, indicating that Gemmeiza is not a favorable environment for those genotypes, while the Sids location was the nearest and more favorable for them (**Figure 1A**). Genotypes TWC324, TWC321, and SC128 were near zero IPCA1, indicating that they had lower GE interaction for disease incidence than other genotypes.

A biplot is made using the genotype and environmental scores from the first two AMMI components. The AMMI 2 biplot provides a graphical illustration of genotype stability and disease incidence ability. The magnitude of genotype–environment interaction is represented by the AMMI biplot, with IPCA1 against IPCA2 (**Figure 1B**). Genotypes TWC368 and TWC324 were the most stable genotypes, characterized by an IPCA1 score near zero. Genotypes Boushy and SC3062 were more favorable for Sids 1 and 3, while genotype TWC360 was more favorable for Sids 2. However, SC130, SC30K8, SC2055, and SC168 were more favorable for Gemmeiza 1 and 3, while SC132, SC30K9, SC2031, SC128, TWC1100, and SC3062 were more favorable for Gemmeiza 2. TWC360 had higher disease incidence than those genotypes at all sites, as indicated by small IPCA1 and IPCA2 scores, which suggests that it was highly unstable (**Figure 1**; **Table 6**).

### AMMI biplot for 100-kernel weights of 15 maize in six environments

**Figure 2:**
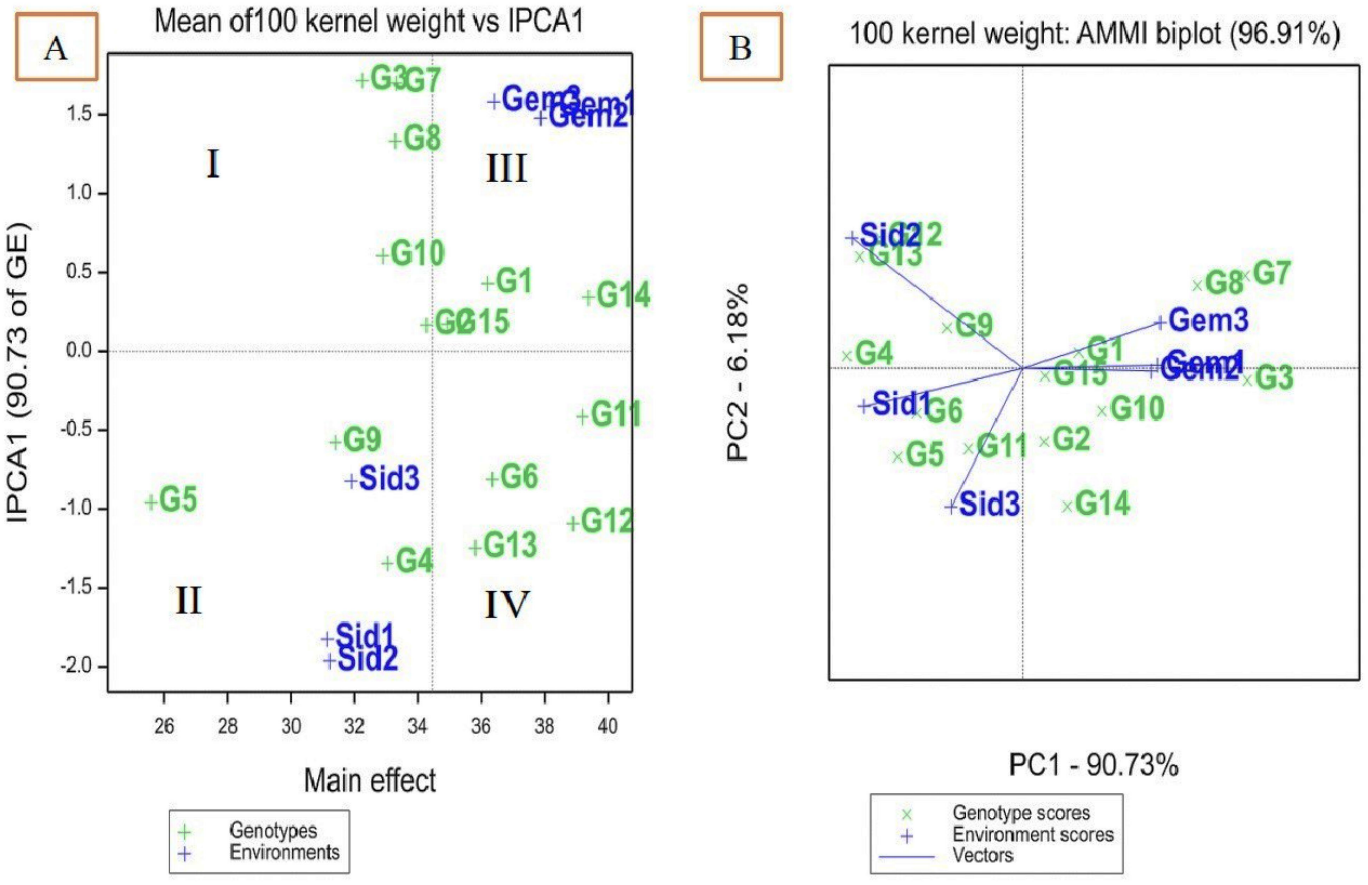
AMMI biplot of interaction principal component axis (IPCA1) against mean effect for 100 kernel weight of 15 maize genotypes in six environments **(A)**, IPCA1 vs. (IPCA2) for 100 kernel weight **(B)**.

In the AMMI 1 biplot (**Figure 2A**), the major effects are represented on the x-axis, while the principal component (IPCA) values are displayed on the y-axis. Genotypes or environments that align closely with a horizontal line exhibit similar interaction patterns, whereas those on a perpendicular line share comparable means. Large IPCA1 scores, whether positive or negative, indicate strong genotype–environment interactions, while genotypes or environments with IPCA1 scores near zero reflect minimal interaction. The biplot is divided into four quadrants (**Figure 2A**). Quadrants I and II represent environments with lower mean 100-kernel weights, while quadrants III and IV contain environments with higher mean 100-kernel weights. Quadrant III includes the ideal environments (Gemmeiza 1 and 2), which are characterized by stable genotypes and high 100-kernel weight. Quadrant IV contains high-yielding but unstable genotypes, quadrant I has stable genotypes with low yields, and quadrant II consists of unstable genotypes with low yields.

The IPCA1 and IPCA2 for 100-kernel weight accounted for 90.73% and 6.18% of the interaction, respectively (**Figure 2B**). The position of a genotype’s vector, extending from the origin (0, 0), indicates its stability relative to the environment. Genotypes with an IPCA1 score of zero are less influenced by environmental variations and exhibit higher adaptability. The genotypes identified as desirable in both **Figures 2A** and B include SC132, TWC360, TWC368, SC30K8, SC128, SC130, TWC1100, and TWC324.

### Anatomical characteristics of maize genotypes

**Figure 3:**
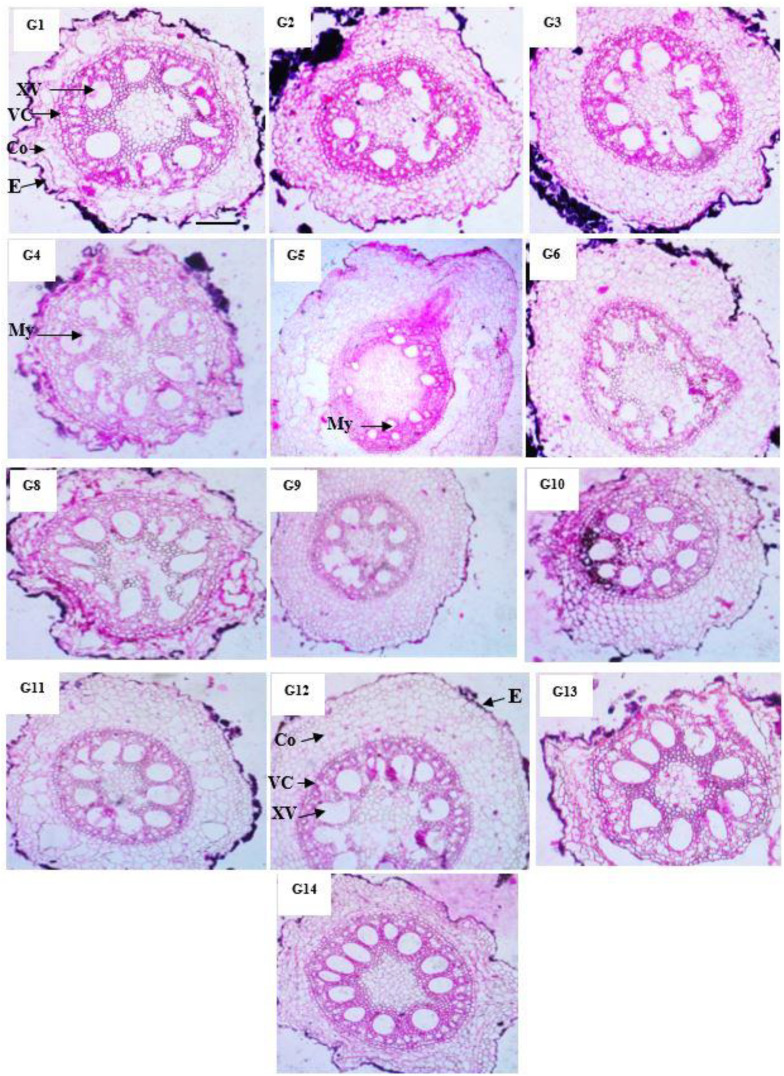
Anatomical structure of maize genotypes under infection with *Magnaporthiopsis maydis*. E, Epidermis; Сo, Cortex; VC, Vascular cylinder; VB, Vascular bundle; XV, Xylem Vessels; My, Mycelium.

Our results in **Table 10** and **Figure 3** indicated that the anatomical traits of maize genotypes were harmfully affected under infection with *M. maydis*. Numerous investigations have detailed alterations in the vascular cylinder as a result of environmental stressors. In plants with roots growing in hypoxic environments, a smaller vascular cylinder would have a greater chance of carrying oxygen, water, and photo assimilates. The anatomical examination showed that mycelial growth was found in the xylem vessels of roots at 45 days under infection with *M. maydis*. The Boushy genotype had the lowest value for all studied anatomical traits. Boushy exhibited anatomical root diameter (307.55), cortex thickness (31.99), vascular cylinder diameter (246.10), vascular bundle diameter (41.74), and number of metaxylem (7). However, genotype SC2031 showed the highest value for most anatomical traits (root diameter 824.09), cortex thickness (169.1), vascular cylinder diameter (491.42), vascular bundle diameter (104.69), and number of metaxylem (11), followed by genotype TWC1100.

**Table 10:** Anatomical characters of Maize genotypes infected with *Magnaporthiopsis maydis*.

Genotype no.	Maize genotype	Anatomical characteristics of maize roots				
		Root diameter	Cortex thickness	Vascular cylinder diameter	Vascular bundle diameter	Number of metaxylem vessels
G1	SC128	711.76	161.6	405.44	67.03	10
G2	SC130	731.29	197.97	441.25	70.01	10
G3	SC132	586.04	98.7	338.28	56.36	8
G4	SC168	568.38	84.1	335.09	54.34	8
G5	Boushy	307.55	31.39	246.1	41.74	7
G6	TWC321	659.15	148.62	356.27	60.6	8
G8	TWC368	595.1	99.99	353.85	57.9	8
G9	SC3062	389.8	57.08	241.95	51	8
G10	SC30K8	622.16	161.6	377.98	67.45	9
G11	SC30K9	749.29	213.24	466.27	81.37	11
G12	SC2031	824.09	169.1	491.42	104.69	11
G13	SC2055	662.47	164.45	424.93	67.92	10
G14	TWC1100	757.63	257.34	490.8	82.25	12

### Expression of the *PR1* and *PR4* genes in maize plants

**Figure 4:**
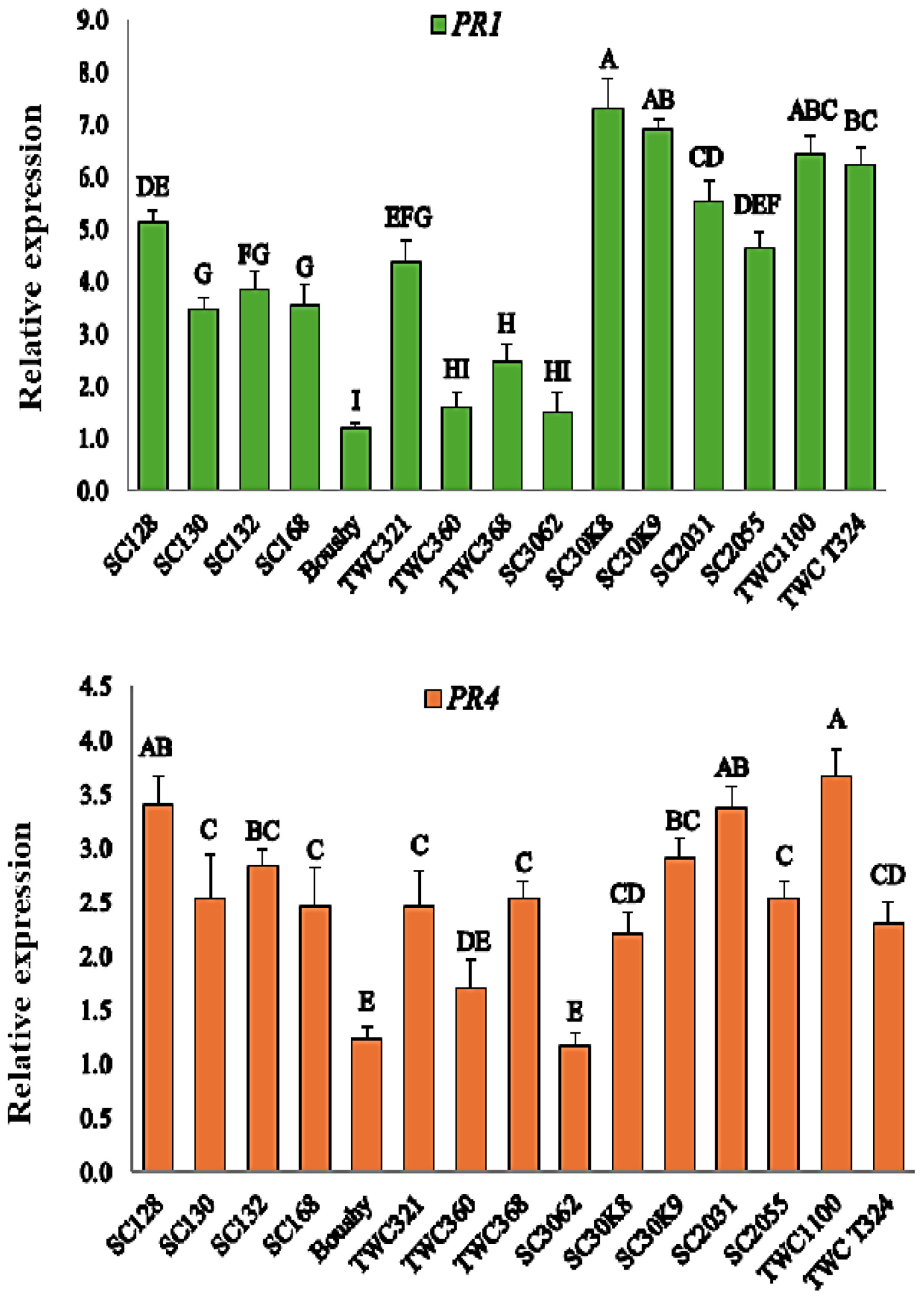
Expression levels of PR1 and PR4 genes in maize plants. Statistically significant differences among treatments are denoted by different letters.

Both the *PR1* and *PR4* genes were elevated under infection with *M. maydis*. Boushy, TWC360, and SC3062 showed the lowest expression levels of *PR1*, whereas SC30K9, SC30K8, TWC1100, TWCT324, and SC2031 showed the highest expression levels after infection (**Figure 4**). *PR4* expression was highly upregulated in TWC1100, SC2031, and SC128 in comparison with other genotypes. In contrast, the expression of *PR4* was downregulated in the Boushy genotype (**Figure 4**).

## Discussion

This study comprehensively evaluates maize genotypes for resistance to late wilt disease caused by *M. maydis*. The results highlight significant genetic variability among the evaluated genotypes, with clear distinctions in their resistance to LWD and yield performance across different environmental conditions. Significant variances between the environments in the current study showed that every environment is different and that the optimal environments must be found to find high-yielding cultivars. Also, the significant differences among the genotypes were due to the differences in genetic makeup and yield traits. However, one of the key challenges in selecting superior maize genotypes under pathogen stress lies in the differential responses of genotypes to various environments. Screening for crop disease resistance requires assessing arrays of genotypes to group genotypes into particular disease response classes (Ohunakin et al., 2019; Abdelghany et al., 2023). Genotypes TWC1100, SC2031, and SC30K9 exhibited low disease incidence and high RRI, according to the epidemiological measures of maize genetic resistance to LWD (DI%, AUDPC, rAUDPC, and RRI). Conversely, the check cultivar Boushy had the lowest RRI and the highest disease incidence. Due to the different genetic makeup of maize genotypes and different environments, different responses to LWD were obtained for genotypes. The best genotypes overall of studied yield traits were SC2031, SC2055, and TWC1100. Because the genetic makeup of maize genotypes varied, there were observed changes in ear yield across locations, leading to significant differences between genotypes (Jeger and Viljanen-Rollinson, 2001). The weight of 100 kernels and ear yield of maize have the most important and intricate quantitative attributes, as several genes control it. Genetic and environmental factors may contribute to variations in maize output in different environments. Ebrahimian et al. (2009) and Ghareeb et al. (2014) reported similar results.

The AMMI model is an effective statistical method for identifying systemic variation in the interaction effect. With the AMMI model, a precise selection of superior genotypes results in a better suggestion of newly developed hybrids, thus enhancing the yield of maize grains in a specific environment. Significant GEI was found for disease incidence and 100-kernel weight of the maize genotypes, indicating the necessity of identifying stable and high-yielding genotypes over years of extensive evaluation in different environments. This result is in agreement with (Moghaddam and Pourdad, 2009; Oyekunle et al., 2017b).

AMMI analysis revealed that the residuals were insignificant, indicating the success of this analysis in partitioning GEI and explaining its components. This suggested that the two first components of genotypes and environment predicted the interaction of maize genotypes with the six environments, which is consistent with the findings of Gauch and Zobel (1996); Agbahoungba et al. (2017), and Shojaei et al. (2021), who suggested that the first two IPCAs can be used to predict the most accurate model for AMMI.

The use of genotype means and overall environments for choosing superior genotypes were diminished for quantitative traits, where there is a significant GEI (Oyekunle et al., 2017a). The most stable genotype in the ASV method is the one with the lowest ASV score (Purchase et al., 2000). The ASV exhibited the most desirable genotypes for the selection of both stability and low disease incidence, which were TWC1100, SC128, TWC368, TWC321, and TWC324 based on the GSI (**Table 6**). The most favorable genotypes for the selection of both stability and high 100-kernel weight were TWC1100, followed by SC30K9, SC130, and TWC324, which had the highest estimated stability parameters and AMMI biplot result. These results agree with those of Farshadfar et al. (2010), who found the least that GSI is considered the most stable with a high 100-kernel weight.

The genotypes and environments with the least stability were positioned farthest from the origin. According to Osiru et al. (2009) and Shrestha et al. (2021), positive interactions occur when genotypes and environments are in the same sector, while negative interactions occur when they are in different sectors. Genotypes SC128 and TWC324 were the most stable, as indicated by their IPCA1 scores close to zero, suggesting minimal interactions with environments and similar disease incidence responses. Purchase (1997) also reported that the most stable genotypes are those closest to the center of the biplot when IPCA1 is plotted against IPCA2.

TWC360 exhibited the highest disease incidence across all sites, as shown by its small IPCA1 and IPCA2 scores, indicating it was highly unstable. Therefore, **Figure 1B** shows that the most stable and low disease incidence genotypes, such as SC30K8, SC2055, TWC324, and TWC1100, were closer to the center of the biplot (Almohammedi et al., 2019; Shrestha et al., 2021). Genotype SC130 exhibited a low disease incidence score with a large IPCA1 score. In contrast, genotypes Boushy, SC3062, and TWC368 were unstable and had high disease incidence (**Figure 1** and **Table 6**).

Genotypes SC132, SC30K8, SC2055, SC30K9, TWC324, TWC1100, and SC128 were better suited for Gemmeiza 1, 2, and 3, while Boushy, SC3062, TWC321, SC168, SC2055, and SC2031 were more suitable for Sids 1, 2, and 3 (**Figure 2A**). Genotypes that show strong positive interactions with specific environments are better adapted to those locations, benefiting from the local agro-ecological and agronomic conditions. Higher yields at a particular location generally arise from positive interaction effects between genotypes and environments with IPCA1 scores of the same sign.

Selecting genotypes for an environment becomes more challenging as the IPCA score for that environment deviates further from zero, indicating greater interaction between the environment and genotypes. To help visually interpret the GEI pattern and identify genotypes or environments with low, medium, or high interaction effects, the AMMI 2 biplot displays the spatial pattern of the first two IPC axes of the interaction effect corresponding to the genotypes (Gauch and Zobel, 1996; Shojaei et al., 2021). **Figure 2B** displays the same desired genotypes (SC132, TWC360, SC30K8, SC30K9, SC128, SC130, TWC324, and TWC1100) in **Figure 2A**. Genotypes that are closer to the origin are considered stable, as they are less influenced by environmental interactions. However, genotypes further from the origin are more affected and show greater interactions. SC130 and TWC324 were the closest to the origin, making them the most stable genotypes (**Figure 2B**). In conclusion, the genotype with the highest yield may not always be the most stable. These results align with the findings of Roseane and Gondim (2012); Oyekunle et al. (2017a), and Shojaei et al. (2021).

Peroxidase activity was significantly increased in the resistant genotypes SC30K9 and TWC324, as compared to the susceptible check cultivar Boushy. The plants possess a standard mechanism to address the accumulation of reactive oxygen species (ROS), which is facilitated by an antioxidant defense system that involves the production of antioxidant enzymes such as catalase (CAT), POD, and PPO (Omara and Abdelaal, 2018; Alafari et al., 2024). Following inoculation, these antioxidant enzymes were activated in the susceptible cultivars, whereas in the resistant cultivars, there was a notable increase in the activities of POD and PPO. These enzymes are crucial for reducing ROS levels, effectively scavenging them to mitigate and eliminate their harmful effects under various stress conditions (Omara et al., 2022, 2023). The EL constitutes an indicator of the increased membrane permeability in the susceptible genotypes than the resistant one. These results may be due to the effect of the pathogen on the cell membrane of the susceptible cultivar and its permeability (Omara and Abdelaal, 2018).

For anatomical traits, the best genotypes overall studied anatomical traits were SC2031 and TWC1100. The thickness of sclerenchyma tissue in the bundle sheath surrounding the vascular bundles in maize stem reflects the tolerance of maize to late wilt disease. Our findings are in agreement with the results of Borkar and Yumlembam (2016) and El-Shabrawy and Shehata (2018) on LWD.

PR genes are involved in various physiological processes and play a central role in plant defense against biotic stress. In recent years, increasing attention has been given to the expression patterns of PR genes as indicators of resistance to pathogen attacks (Esmail et al., 2022). In this study, of the 15 genotypes, Boushy exhibited limited or no expression of the *PR1* and *PR4* genes, explaining the result that Boushy exhibited the highest disease incidence and the lowest *RR1*. Interestingly, *PR1* and *PR4* were highly expressed in TWC1100, which showed also a good result in late wilt resistance. The analysis revealed that the *PR1* and *PR4* genes could perform important functions in maize resistance to LWD. This suggests that *PR1* and *PR4* may play significant roles in maize defense against *M. maydis*. These genes are known to be regulated by defense-related plant hormones such as salicylic acid (SA), abscisic acid (ABA), and methyl jasmonate (MeJA), which may explain their involvement in disease resistance mechanisms (ElSharkawy et al., 2022). Moreover, transcriptomic data have shown that PR1 genes are upregulated in maize following infection with various pathogens, including *Sporisorium reilianum*, *Sesamia nonagrioides*, *Fusarium moniliforme*, *Glomerella graminicola*, and *Phytophthora cinnamomi* (Ma et al., 2022), supporting their broad role in plant defense.

In summary, the integration of agronomic performance, anatomical characteristics, biochemical responses, and gene expression profiles offers a robust approach for selecting and breeding maize genotypes that combine yield stability with strong resistance to late wilt disease across varying environments.

## Conclusion

This study provided a multi-dimensional assessment of 15 maize genotypes for resistance to LWD caused by *M. maydis*, integrating agronomic, anatomical, biochemical, and molecular traits. Significant genetic variability was observed among genotypes across six environments. Genotypes TWC1100, SC2031, and SC30K9 consistently exhibited strong resistance, stable yields, and favorable physiological and molecular profiles. The study identified the most stable genotypes and ideal environments for high resistance and productivity. The AMMI biplot facilitated the visualization of genotype–environment interactions and supported selection decisions. Based on AMMI stability value (ASVi) and genotype selection index (GSIi), SC128, TWC1100, and TWC324 were identified as stable genotypes with low disease incidence and high 100-kernel weight. The AMMI biplot indicated that SC132, TWC360, TWC368, SC30K8, SC128, SC130, and TWC1100 were well-suited to Gemmeiza 1–3, while SC30K9, SC3062, TWC321, SC168, SC2055, and SC2031 were better adapted to Sids 1–3. The AMMI 1 model further revealed that SC130, TWC321, TWC368, and TWC324 performed well across all environments. Notably, the upregulation of the *PR1* and *PR4* genes, along with elevated levels of antioxidant enzymes, was strongly associated with enhanced resistance, indicating that both constitutive and inducible defense responses are critical in mitigating LWD severity. Additionally, anatomical features such as increased sclerenchyma thickness in the stem contributed to mechanical resistance against pathogen invasion. These findings highlight the complexity of LWD resistance and underscore the importance of selecting genotypes that combine high yield potential with durable resistance mechanisms, offering valuable insights for breeding climate-resilient maize and advancing sustainable disease management strategies.

## Data Availability

The raw data supporting the conclusions of this article will be made available by the authors, without undue reservation.
